# Perceptions and Expectations of School-Based Professionals Surrounding School-Based Mindfulness Training in Appalachia During the COVID-19 Pandemic: A Qualitative Study

**DOI:** 10.3389/fpubh.2022.816494

**Published:** 2022-02-04

**Authors:** Ilana Haliwa, Hannah Layman, Jessica Coffman, Amy Snodgrass, Pamela Santer, Brittney Barlett, Kate Long, Ashley Mason, Gretchen Pfost, Jenny Harden, Geri Dino, Traci Jarrett

**Affiliations:** ^1^Department of Psychology, West Virginia University, Morgantown, WV, United States; ^2^WVU School of Public Health, West Virginia Prevention Research Center, Morgantown, WV, United States; ^3^Department of Rural Health, West Virginia University, Morgantown, WV, United States; ^4^Wellness Center, WVU Parkersburg, Parkersburg, WV, United States; ^5^Try This West Virginia, Charleston, WV, United States; ^6^School of Physical Therapy, Marshall University, Huntington, WV, United States; ^7^Greenbrier County Schools, Rupert, WV, United States

**Keywords:** mindfulness, COVID-19, Appalachia, compassion fatigue, burnout

## Abstract

**Background:**

School-based professionals often report high burnout, particularly in geographic areas like Appalachia, where school-aged children are exposed to high levels of adverse childhood experiences, which may be exacerbated by the COVID-19 pandemic. While school-based mindfulness trainings can reduce burnout, their efficacy is influenced by the expectations of intervention personnel ahead of implementation. The present study assessed expectations and perceptions of a school-based mindfulness training among school personnel in 21 Appalachian schools during the COVID-19 pandemic.

**Methods:**

Upon enrollment in the training, staff (*N* = 191) responded to open ended survey questions regarding perceived impacts of COVID-19 on students, expected benefits and barriers to school-based mindfulness, and perceived community acceptance of mindfulness.

**Results:**

School personnel identified social isolation and lack of structure as negative impacts of COVID-19 on students. Expected benefits of classroom mindfulness included improved coping skills, focus, and emotion regulation, whereas barriers included lack of time and student ability level (e.g., age, attention). While most respondents indicated that their community was accepting of mindfulness practices, some noted resistance to and misperceptions of mindfulness, which may illustrate the influence of local cultural norms and values on the acceptability of mental health interventions.

**Conclusions:**

Overall, these findings suggest positive expectations and relative perceived support for mindfulness practices within these Appalachian communities, including in response to negative impacts of the COVID-19 pandemic on students. Adapting practices and language to accommodate barriers such as time, student ability, and cultural misconceptions of mindfulness may increase the feasibility and efficacy of these interventions.

## Introduction

School-based professionals, such as classroom teachers and school staff, play a critical role in both the education and social-emotional development of their students. Unfortunately, school personnel often report high levels of stress and burnout (1–3) that contribute to high turnover rates (4, 5). Appalachia, a geographic and cultural region in the Eastern United States surrounding the Appalachian Mountains (6), provides alarming statistics in this regard. For example, in West Virginia, the only state fully within Appalachia, up to 32% of first-time teachers leave the profession in their first 4 years (7). In West Virginia and Kentucky, an average of 9–10% of all teachers leave the profession, annually (7, 8).

One critical reason for this high turnover rate is compassion fatigue, a form of burnout that is heavily documented among persons in “helping” professions, such as teachers (9, 10). Compassion fatigue involves first- or second-hand exposure to the trauma of others, including teaching or counseling students with Adverse Childhood Experiences (ACEs; e.g., not meeting basic needs due to low family income, divorce, or separation of a parent or guardian). This may be of particular concern in Appalachia, a region that is largely rural, and where regional household income is significantly lower than the national average (11), as rurality and low-income have been associated with higher likelihood of experiencing ACES (12–15).

Moreover, stress related to the current COVID-19 pandemic may exacerbate the impact of ACEs in Appalachian communities, due to factors such as decreased access to basic services, heightened risk of domestic violence from caregivers, social isolation, and economic challenges (16). Widespread classroom closures during the early stages of the pandemic are also predicted to have long-term consequences for the wellbeing of school-aged children, particularly those from disadvantaged or distressed populations (17, 18). Finally, re-introduction to the classroom following an extended period of remote learning may also represent a source of distress for both students and school personnel (19–21). As such, Appalachian school personnel may experience increased compassion fatigue and burnout due to increased primary and secondary trauma both during and in the aftermath of the pandemic.

Some trainings, such as school-based mindfulness, may have the potential to attenuate burnout in the face of these heightened personal and secondary experiences of trauma. School-based mindfulness involves the use of exercises (e.g., physical movement, guided breathing) to increase mindfulness, defined as the non-judgmental attention to the present moment (22), among students and school personnel (23, 24). While mindfulness is associated with lower burnout among teachers, staff, and administrators (25) and school-based mindfulness trainings have been shown to reduce burnout and compassion fatigue among teachers (24, 26–28), little research exists within the context of a global pandemic.

As with many school-based interventions, perceptions of programming are critical in determining the acceptability and effectiveness of implementation (29–31). For example, one study found that teachers who endorsed more positive perceptions of a school-based social-emotional training reported greater implementation fidelity [i.e., dosage and quality of delivery; (31)], which is a significant predictor of program effectiveness (32, 33). Interviews with school-based intervention developers (30), teachers, and students (29) have also identified positive expectations and perceptions (or, “buy-in”) of intervention administrators as a key component to successful intervention delivery and impact. Expectations and perceptions of school-based mindfulness may be influenced by cultural contexts. In areas such as Appalachia certain community characteristics, such as resistance or mistrust of new people or ideas, may be a barrier to implementation of contextually novel or alternative programming, such as mindfulness training (34, 35). Additionally, perceptions of school-based mindfulness trainings may be further influenced by the context of the COVID-19 pandemic. For example, though school personnel may perceive increased potential benefits from interventions to reduce burnout, there are also unique barriers to implementation, including challenges associated with altering program delivery to fit virtual or hybrid classrooms, social distancing guidelines, and abbreviated school hours. The purpose of this study was to identify the perceived need for school-based intervention as a result of the pandemic in Appalachia, as well as the expected benefits and barriers to school-based mindfulness interventions among school personnel, in order to inform tailoring of future interventions to promote support and buy-in, thereby increasing the chances of intervention success.

## Method

In fall 2020 and winter 2021, school personnel across 21 West Virginia elementary schools and two pre-schools were selected to participate in an online training to implement Kidding Around Yoga, a school-based mindfulness-based approach to yoga, as part of two grant-funded efforts (36). In additional to the physical practice of yoga, Kidding Around Yoga incorporates mindfulness practices including breathing exercises, mindful walking, and meditation. This program is aimed at helping teachers lower stress and anxiety and promote resilience in the children and themselves. The West Virginia Prevention Research Center partnered with the funded organizations to conduct an evaluation of the program implementation and outcomes. This study was reviewed and approved by West Virginia University's Institutional Review Board (Approval # 2006044813). Electronic informed consent to participate in this study was obtained from all participants.

### Training

Due to COVID-19 restrictions, all trainings were offered online. Two types of Kidding Around Yoga training programs were offered, EduKAY and OKAY. EduKAY is a 6-h training led by a live online instructor that provides teachers with the skills to implement the Kidding Around Yoga curriculum in their classroom. EduKAY includes techniques to introduce mindful physical activity, yoga, meditation, and mindfulness in general into academic settings. The OKAY certification program is a combination of live sessions and self-paced video series that can be completed up to 5 months after participants are registered. To become certified, participants must submit a video recording of themselves teaching a class and must attend a live 4-h workshop session.

### Recruitment

Schools from both urban and rural communities in West Virginia were invited to participate in the training. Schools for each of the two participating grants were identified either by (1) the level of economic distress of their student population, as specified by the grant or, (2) *via* a statewide listserv of schools, Facebook groups, direct school outreach, and school partners.

### Data Collection

Prior to program implementation, school personnel were invited to participate in an online survey to assess barriers and potential facilitators of conducting mindfulness programming in schools, in general. Email invitations and up to three reminders were sent to 335 participants who enrolled in the online Kidding Around Yoga trainings. Of that number, 191 consented and completed a pre-intervention survey (response rate 57%) in which they responded to open-ended questions about perceived benefits and barriers to engaging in the use of mindfulness techniques in school settings and community perceptions of mindfulness (See Table 1).

**Table 1:** Open-ended survey questions.

**COVID-19 impact**
“*COVID-19 has changed the educational landscape. What do you think is the biggest impact on your students and their families as a result of COVID-19?”*
**Expected benefits**
“*What do you think the benefits of mindful practice and/or mindful classrooms might have on you personally?”*
“*What do you think the benefits of mindful practice and/or mindful classrooms might have on your students?”*
**Expected barriers**
“*Do you anticipate barriers to practicing mindfulness in your classroom? If so, what do you think they will be?”*
**Community acceptance**
“*Do you feel like mindfulness practice is accepted in your community? Why or why not?”*

Responses were analyzed using thematic qualitative analysis (37). First, all three reviewers reviewed responses to each open-ended survey question and performed inductive coding, identifying core themes for each question. This round of coding was followed by a thorough reading by the primary author in which each theme was examined for consistency and divergence from its operational definition. Coding rules based on the operational definitions for each category were developed, and responses were independently coded based on those definitions by three independent coders. In the results, we provide the percentage of participants who endorsed each theme (i.e., the number of participants who endorse the theme out of the total number of participants who responded to the survey question). We also report percent agreement across independent coders, as calculated by the number of instances of agreement on coding (i.e., statements coded consistently across reviewers) out of total items coded for each theme. Percent agreement across themes ranged from 90.10 to 100%.

## Results

**Figure 1:**
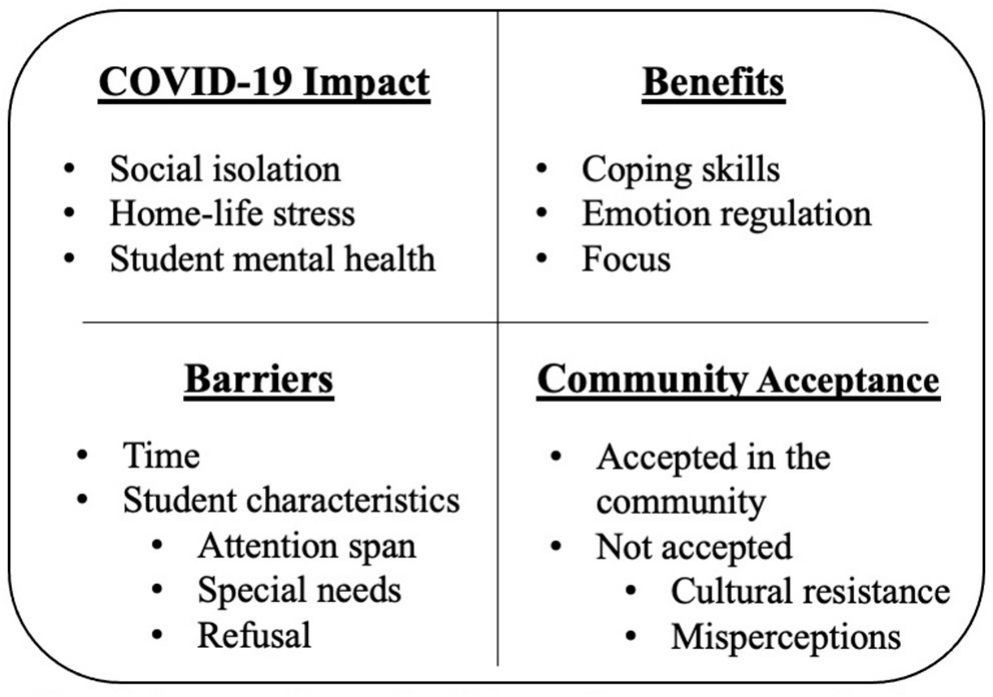
Summary of themes identified across four response categories: (1) COVID-19 impact, (2) expected benefits, (3) expected barriers to school-based mindfulness, and (4) community acceptance of mindfulness.

This study sought to identify elementary school personnel's perceived need for school-based mindfulness in the context of the COVID-19 pandemic, and the expected benefits and barriers associated with this programming. Participants were 191 school personnel enrolled in the online Kidding Around Yoga training. The majority were female (95.2%; 4.3% male, 0.5% preferred not to answer) and White (94.1%; 1.6% Black/African American; 0.5% Asian; 0.5% preferred not to answer; 3.2% other, or more than one race). The majority of participants had 7 or more years of education experience (68.3%; 9.8% 5–6 years; 11.5% 3–4 years; 4.9% 1–2 years; 5.5% <1 year) and time spent working in the school system was varied (16.6% <1 year; 18.2% 1–2 years; 20.9% 3–4 years; 13.4% 5–6 years; 31.0% 7+ years). Overall, we identified response themes across four domains: (1) perceived impact of COVID-19 on students, (2) anticipated benefits of school-based mindfulness training, (3) anticipated barriers to school-based mindfulness training, and (4) perceived community acceptance of mindfulness practices (See Figure 1).

### COVID-19 Impact

Participants were asked to describe what they perceived as the most significant impacts of the COVID-19 pandemic on students and their families (123 respondents). Overall, school personnel reported concerns that their students were suffering from social isolation, exacerbated home-life stressors, and worsened mental health. The most cited impact of the pandemic, overall, was social isolation of students and their families (40.65% of respondents, 91.06% agreement). This included social isolation of students from their peers (“*Limited amount of social interaction that has made it harder for students to relate to each other*.”), as well as social isolation within the home for students from distressed families. For example, some participants highlighted a “*lack of in-person socialization and touch… many students' home lives are not the best and these students have really struggled in many aspects while being quarantined.”*

In addition to social isolation, respondents perceived that home-life stressors were another significant impact of the pandemic on their students (25.20% of respondents, 91.06% agreement). These stressors included lack of resources (“*I have students who have families losing employment, working out of state, and one without basic needs.”*) and greater parental stress (“*The children are in school less and home more. This causes stress on parents who are having trouble getting their children to learn.”)*. These added familial stressors, along with existing histories of abuse were additional concerns (“*Students that are victims of abuse and neglect are going unnoticed since they are not physically at school. I am worried about the impact socially, mentally, physically, and emotionally on our students.”*).

Finally, respondents were also concerned about worsened mental health across their students (13.82% of respondents; 91.87% agreement). Concerns centered around the above stressors as well as general fear of the unknown, negatively impacting students' wellbeing overall.

### Expected Benefits

In order to determine what benefits participants expected from training, and to determine whether these were salient with respect to the aforementioned impacts of COVID-19 on students, participants were asked three separate questions in which they were prompted to describe expected benefits of incorporating school-based mindfulness for (1) themselves (123 respondents), (2) their students (126 respondents), (3) and in the context of the COVID-19 pandemic (120 respondents). Improved ability to cope with distress emerged as a primary theme across responses, with respect to personal ability to cope (53.66% of respondents, 99.19% agreement), student ability to cope (57.14% of respondents, 91.34% agreement), and ability to cope specifically within the context of the COVID-19 pandemic (41.67% of respondents, 91.67% agreement). Participants highlighted expecting mindfulness training to promote feelings of calmness, clarity, and positive cognition (“*Mindfulness allows my mind to calm down. It allows me to think more clearly and appropriately. It provides a more peaceful day with more positive thoughts*.”). Further, participants largely emphasized that the training would benefit themselves and their students by providing coping tools for stressful situations both within the classroom and those outside of the classroom (“*I believe it will provide them [students] with strategies to work through often stressful, traumatic situations even outside of the classroom.”*). These benefits were expected to extend to the ability to cope with the stress of the COVID-19 pandemic (“*Mindful practice will help my students cope with COVID. Teaching them how to deal with their inner emotions and feelings will give them strategies to cope with how they are feeling.”*).

Though mentioned less consistently, improved student emotion regulation (30.95% of respondents, 95.28% agreement) and student focus (22.22% respondents, 97.64% agreement) emerged as two additional themes throughout participants' responses. Respondents also associated greater emotion regulation and ability to focus to improve student behavior and academic performance (“*… Mindfulness can help improve attention and focus, which will aid in better grades. Giving students a guide to help them regulate their emotions and generate better behavior in school.”*).

### Expected Barriers

Participants were also asked to describe perceived barriers to school-based mindfulness (125 respondents). The most common response to this question was that, upon enrollment, participants did not anticipate any barriers (40.80% of respondents, 96.00% agreement). Of those who did perceive potential barriers, time was identified as a significant barrier (20.80% of respondents, 98.40% agreement). For example, one participant noted that “*…time constraints may be the biggest barrier in the general classroom setting.”* For some respondents, these time constraints were exacerbated by changes to the school schedule during the COVID-19 pandemic such as reduced hours (“*Space and time. We are right now in hybrid so teachers feel the need to use this time to get as much done because we only see the students 2 times a week.”*).

Finally, certain student characteristics, such as age, attention span, ability-level, and reluctance to participate (19.20% of respondents, 99.20% agreement) were also identified as potential barriers to school-based mindfulness. Specifically, participants were concerned that younger students and students with special needs would struggle to engage with mindfulness practices within the classroom (“*… I have 4- and 5-year-olds. I also have special needs students. Their attention spans are very short. I could apply them [mindful classroom practices] but only in short forms*.”). In addition, some participants were unsure that their students would be willing to participate in the exercises due to feelings of vulnerability and uncertainty about the subject (“*I feel the biggest barrier to practicing mindfulness in my classroom would be my students feeling vulnerable. Many students are not used to the concept of mindfulness and are probably uncertain of how to feel about it.”*).

### Community Acceptance

Participants responded to an open-ended question about whether they perceived their community to be accepting of mindfulness practices (121 respondents). Of respondents, 42.99% perceived that their community *was* accepting of mindfulness practice (94.22% agreement). Participants specifically mentioned the support of parents and school communities due to their shared understanding of students experience of trauma (“*Yes. I think our community and school system is very aware and supportive of mental health and mindfulness practices to help our students because we have such a large amount of students who come from traumatic experiences/households.”*).

However, 28.10% of respondents (90.10% agreement) reported a lack of support for mindfulness practice within their community. Some of these respondents attributed this lack of support to misperceptions or misunderstanding of mindfulness practices (“*I feel like much of our community sees it as a waste of time and some “hippie” program.”*). Community resistance to certain contextually novel concepts, like mindfulness, were also cited as reasons for a lack of community support (“*It is a very conservative community that may be hesitant to accept practices that are not considered western.”*). This resistance was attributed by some to the uniquely rural and conservative nature of Appalachia itself (“*Probably not- we are a rural, Appalachian community. Anything “new” or “different” isn't easily trusted.”*).

## Discussion

This study used qualitative data analysis to understand perceived impacts of the COVID-19 pandemic on students in Appalachia, as well as expectations and perceptions of a school-based mindfulness training and community acceptance of mindfulness, among staff across 21 Appalachian schools. School-based mindfulness techniques have the capacity to help both staff and students cope with stressful life experiences, including those related to COVID-19, and prevent or reduce burnout (24, 26–28). These practices may be particularly helpful in rural areas where the prevalence of ACES is relatively high (12–14) and may be exacerbated by certain global stressors, such as the COVID-19 pandemic (16). Given that expectations and buy-in are critical predictors of the success of school-based programming (29–31), it is crucial to understand expectations and perceptions related to mindfulness within targeted communities to maximize acceptability, feasibility, and implementation.

Overall, school personnel reported three main concerns regarding the impacts of the COVID-19 pandemic on students: social isolation, exacerbation of home-life stressors, and worsened mental health. This is consistent with early research pointing to exacerbation of ACES during the pandemic, including stress related to social isolation (38, 39), exacerbation of home-life stressors (40, 41) and worsened mental health (42, 43) among school-aged children due to the pandemic. Further, as the effects of the pandemic are hypothesized to be intensified for individuals already at risk for ACES (i.e., those with low-income, individuals in rural communities), Appalachian students may represent a particularly high-risk population for the development or worsening of ACES due to the pandemic (11–15). Given this increased risk for Appalachian students, it holds that school personnel may bear the burden of increased risk of burnout and compassion fatigue as they return to interacting with and supporting these students. As such, incorporation of school-based interventions that have been shown to attenuate burnout and support student mental health will be critical.

Promisingly, participants generally reported positive expectations with respect to school-based mindfulness. Expectations included greater ability to cope with distress for both school personnel and their students, both generally and specific to pandemic related distress. Additionally, respondents expected additional benefits for their students because of school-based mindfulness, including improved emotion regulation and focus. This is consistent with existing evidence demonstrating improvements in coping strategies, emotion regulation, and focus among students and school personnel following implementation of school-based mindfulness interventions (24–28).

In addition to expected benefits, participants also reported some expected barriers to school-based mindfulness. Respondents reported that time was a significant barrier to implementing school-based mindfulness, particularly given reduced in-person interaction with students due to the COVID-19 pandemic. Further, certain student characteristics were identified as potential barriers to school-based mindfulness, including age, attention span, ability level, and apprehension or refusal. Thus, these may be important considerations to incorporate into school-based mindfulness trainings. For example, brief practices that can be easily implemented both virtually and in-person throughout the school day may circumvent time barriers, including those related to COVID-19. Indeed, existing evidence suggests the efficacy of mindfulness practices as brief as 5-min long (44). Further, it may be critical to introduce mindfulness practices accessible to a wide range of age and ability levels and evidence-based mindfulness practices are available for children as young as pre-school age and for a variety of ability levels (26, 45, 46). Information and education about the availability and effectiveness of these adaptations might be valuable to include in future trainings.

Survey responses also suggest that almost half of respondents perceived mindfulness practices to be accepted within their community. These findings are promising and suggest that, given exacerbated experiences of trauma and burnout within these regions during the COVID-19 pandemic, Appalachian schools may be fertile ground for incorporating mindfulness as a tool to help school personnel and students improve their coping skills, increase emotion regulation, and focus, and attenuate burnout. Despite this, 28.10% of respondents did suggest that mindfulness practices were *not* accepted within their community. Common reasons included misunderstanding of mindfulness as a practice (e.g., perceived as “hippie” or not in alignment with western religion) and cultural resistance to new or different practices. These results are consistent with prior research suggesting that communities in rural Appalachia may be initially distrusting or suspicious of novel or unfamiliar interventions (34, 35). However, school-based mindfulness may be particularly beneficial in rural Appalachian communities, given their disproportionately high levels of ACES and teacher burnout (7, 8, 12–14). As such, it may be necessary to adapt some classroom mindfulness training and intervention language to promote greater acceptability within these communities. For example, including educational materials for parents may help to prevent misunderstanding and dispel myths surrounding mindfulness practice. Adaptation of trainings using language that is more familiar and accessible (e.g., using terms such as “relaxation” and “stress relief” rather than “mindfulness”; or “focused breathing” rather than “meditation”) may also be beneficial in easing suspicion of novel interventions.

The present study is not without limitations. While qualitative coding of data allows the researchers to understand various dimension of participant experience, including perceived benefits and barriers, additional research could incorporate quantitative assessments of changes across many of the themes mentioned throughout the present survey responses, including mental health and burnout, using validated scales. Further, data for the present study was collected largely during the fall of 2020, a unique time-period during which many schools were operating remotely due to the COVID-19 pandemic. Thus, while the present findings provide unique insight into perceptions of a school-based mindfulness training in Appalachia during a time of significant stress for teachers and staff, some of these findings may not generalize to post-pandemic times (i.e., COVID-19 related barriers to implementation). Finally, responses were collected from staff at schools that had already accepted funding to conduct school-based mindfulness trainings, and thus responses may be skewed to reflect more favorable responses and perceptions. However, findings from the present study provide an initial understanding of perceptions and expectations related to school-based mindfulness within these unique Appalachian communities. Future research studies can build upon these findings by incorporating quantitative assessments of change across these identified themes using validated scales and by replicating this study within samples of school-based professionals who have *not* already agreed to participate in a mindfulness training.

In summary, school personnel reported concerns about their students in relation to social isolation, worsened home-life stressors, and decreases in mental health due to the COVID-19 pandemic. Participants generally anticipated school-based mindfulness to be beneficial for themselves and their students, in improving coping skills, emotion regulation, focus, and attenuating related effects of the pandemic. However, lack of time and student ability-levels were identified as potential barriers. While many respondents reported that their community was accepting of mindfulness, some were less confident, citing cultural resistance to novel practices and misperceptions of mindfulness. Taken together, these findings suggest that Appalachian school communities are promising candidates for school-based mindfulness interventions, given positive expectations and relative perceived support for mindfulness practices within the community. Incorporating shorter mindfulness practices accessible to a range of ability levels and adapting trainings to use language that is more familiar and accessible within communities may increase the feasibility and efficacy of these interventions within schools.

## Data Availability Statement

The raw data supporting the conclusions of this article will be made available by the authors, without undue reservation.

## Ethics Statement

The studies involving human participants were reviewed and approved by West Virginia University Institutional Review Board. The patients/participants provided their written informed consent to participate in this study.

## Author Contributions

BB, KL, AM, and GP were instrumental in funding and implementation of study programming. TJ organized the database. IH, HL, and JC performed the statistical analysis. IH wrote the first draft of the manuscript. HL and TJ wrote sections of the manuscript. All authors contributed to conception and design of the study, manuscript revision, read, and approved the submitted version.

## Funding

Research in this publication was supported by a grant from the U.S. Department of Health and Human Services, Substance Abuse and Mental Health Services Administration [State Opioid Response (SOR) 1H79TI081724-01)] administered through the West Virginia Department of Health and Human Resources Bureau for Behavioral Health and the WV School for Osteopathic Medicine.

## Conflict of Interest

The authors declare that the research was conducted in the absence of any commercial or financial relationships that could be construed as a potential conflict of interest.

## Publisher's Note

All claims expressed in this article are solely those of the authors and do not necessarily represent those of their affiliated organizations, or those of the publisher, the editors and the reviewers. Any product that may be evaluated in this article, or claim that may be made by its manufacturer, is not guaranteed or endorsed by the publisher.

## References

[1] Combs J Edmonson SL Jackson SH. Burnout among elementary school principals. AASA J Scholarsh Pract. (2009) 5:10–5.10431286

[2] Koenig A Rodger S Spect J. Educator burnout and compassion fatigue: a pilot study. Canad J School Psychol. (2018) 33:259–78. 10.1177/0829573516685017

[3] McCormick J Barnett K. Teachers' attributions for stress and their relationship with burnout. Int J Educ Manag. (2011) 25:278–93. 10.1108/09513541111120114

[4] Ingersoll R. Teacher turnover and teacher shortages: an organizational analysis. Am Educ Res J. (2001) 38:499–534. 10.3102/00028312038003499

[5] National Center for Education Statistics. The Condition of Education (2016). (2016). Available online at: https://nces.ed.gov/pubs2016/2016144.pdf (accessed January 10, 2022).

[6] Appalachian Regional Commission,. About the Appalachian Region. (2022). Available online at: https://www.arc.gov/about-the-appalachian-region/ (accessed January 10, 2022).

[7] Lochmiller CR Adachi E Chesnut CE Johnson J. Teacher Retention, Mobility, and Attrition in West Virginia. Washington, DC: US Department of Education, Institute of Education Sciences, National Center for Education Evaluation and Regional Assistance, Regional Educational Laboratory Appalachia (2016).

[8] Lochmiller CR Sugimoto TJ Muller PA. Teacher Retention, Mobility, and Attrition in Kentucky Public Schools From 2008 to 2012 (REL 2016–116). Washington, DC: US Department of Education, Institute of Education Sciences, National Center for Education Evaluation and Regional Assistance, Regional Educational Laboratory Appalachia (2016).

[9] Hill R Vaccarino O Daly KJ. Understanding Compassion Fatigue. Guelph, ON: University of Guelph Atrium (2015).

[10] Lewis ML King DM. Teaching self-care: the utilization of self-care in social work practicum to prevent compassion fatigue, burnout, and vicarious trauma. J Hum Behav Social Environ. (2019) 29:96–10. 10.1080/10911359.2018.1482482

[11] Pollard K Jacobsen LA. The Appalachian Region: A Data Overview From the 2014–2018. American Community Survey (2020). Available online at: https://assets.prb.org/pdf20/prb-arc-chartbook-2020.pdf (accessed January 10, 2022).

[12] Crouch E Probst JC Radcliff E Bennett KJ McKinney SH. Prevalence of adverse childhood experiences (ACEs) among US children. Child Abuse Neglect. (2019) 92:209–18. 10.1016/j.chiabu.2019.04.010 31003066

[13] Hartley D. Rural health disparities, population health, and rural culture. Am J Public Health. (2004) 96:1675–8. 10.2105/AJPH.94.10.1675 15451729 PMC1448513

[14] Leider JP Meit Mm McCullough JM Resnick B Dekker D Bishai D. The state of rural public health: Enduring needs in a new decade. Am J Public Health. (2020) 110:1283–90. 10.2105/AJPH.2020.305728 32673103 PMC7427223

[15] Walsh F McCartney G Smith M Armour G. Relationship between childhood socioeconomic position and adverse childhood experiences (ACEs): a systematic review. J Epidemiol Commun Health. (2019) 73:1087–93. 10.1136/jech-2019-212738 31563897 PMC6872440

[16] Bryant DJ Oo M Weitman I. The rise of adverse childhood experiences during the COVID-19 pandemic. Trauma Psychol. (2020) 12:S193–4. 10.1037/tra0000711 32551773

[17] Eyles A Gibbons S Montebruno P. COVID-19 School Shutdowns: What Will They Do to Our Children's Education? (2020). Available online at: http://eprints.lse.ac.uk/104675/3/Eyles_covid_19_school_shutdowns_published.pdf (accessed January 10, 2022).

[18] UNICEF Innocenti Research Center Borokowski A Ortiz-Correa JS Bundy DAP Burbano C Hayashi C. COVID-19: Missing More Than a Classroom. Florence. (2021). 10.18356/25206796-2021-01

[19] Capurso M Dennis J Salmi L Parrino C Mazzeschi C. Empowering children through school re-entry activities after the COVID-19 pandemic. Contin Educ. (2020) 1:64–82. 10.5334/cie.17 PMC11104315 38774523

[20] Levinson M Cevik M Lipsitch M. Reopening primary schools during the pandemic. N Engl J Med. (2020) 383:981–5. 10.1056/NEJMms2024920 32726550

[21] Pelaez M Novak G. Returning to school: separation problems and anxiety in the age of pandemics. Behav Anal Pract. (2020) 13:521–6. 10.1007/s40617-020-00467-2 32837704 PMC7362323

[22] Kabat-Zinn J. Wherever You Go, There You Are: Mindfulness Meditation in Everyday Life. New York, NY: Hyperion (1994).

[23] Greenberg MT Harris AR. Nurturing mindfulness in children and youth: current state of research. Child Dev Persp. (2012) 6:215. 10.1111/j.1750-8606.2011.00215.x30738238

[24] Meiklejohn J Phillips C Freedman ML Griffin ML Biegel G Roach A. Integrating mindfulness training into K-12 education: Fostering the resilience of teachers and students. Mindfulness. (2012) 3:291–307. 10.1007/s12671-012-0094-5

[25] Abenavoli RM Jennings PA. Greenberg, MT, Harris AR, Katz DA. The protective effects of mindfulness against burnout among educators. Psychol Educ Rev. (2013) 37:57–69.

[26] Flook L Goldberg SB Pinger L Bonus K Davidson RJ. Mindfulness for teachers: a pilot study to assess effects on stress, burnout and teaching efficacy. Mind Brain Educ. (2013) 7:12026. 10.1111/mbe.12026 24324528 PMC3855679

[27] Donahoo LMS Siegris B Garrett-Wright D. Addressing compassion fatigue and stress of special education teachers and professional staff using mindfulness and prayer. J School Nurs. (2018) 36:442–8. 10.1177/1059840517725789 28812432

[28] Sun J Wang Y Wan Q Huang Z. Mindfulness and special education teachers' burnout: the serial multiple mediation effects of self-acceptance and perceived stress. Social Behav Pers. (2019) 47:e8656. 10.2224/sbp.8656

[29] Dariotis JK Mirabal-Beltran R Cluxton-Keller F Gould LF Greenberg MT Mendelson T. Qualitative exploration of implementation factors in a school-based mindfulness and yoga program: lessons learned from students and teachers. Psychol Sch. (2017) 54:53–69. 10.1002/pits.21979 28670007 PMC5486971

[30] Forman SG Olin SS Hoagwood KE Crowe M Saka N. Evidence-based interventions in schools: developers' views of implementation barriers and facilitators. School Mental Health. (2009) 1:26–36. 10.1007/s12310-008-9002-5

[31] Ransford CR Greenberg MT Domitrovich CE Small M Jacobson L. The role of teachers' psychological experiences and perceptions of curriculum supports on the implementation of a social and emotional learning curriculum. School Psychol Rev. (2009) 38:510–32.

[32] Bellg AJ Borrelli B Resnick B Hecht J. Minicucci DS Ory M. Enhancing treatment fidelity in health behavior change studies: Best practices and recommendations from the NIH behavior change consortium. Health Psychol. (2004) 23:443–51. 10.1037/0278-6133.23.5.443 15367063

[33] Breitenstein SM Gross D Garvey C Hill C Fogg L Resnick B. Implementation fidelity in community-based interventions. Res Nurs Health. (2010) 33:164–73. 10.1002/nur.20373 20198637 PMC3409469

[34] Behringer B Friedell GH Dorgan KA Hutson SP Naney C Phillips A. Understanding the challenges of reducing cancer in Appalachia: addressing a place-based health disparity population. Cal J Health Promot. (2007) 5:40–9. 10.32398/cjhp.v5iSI.1197

[35] Deskins S Harris CV Bradlyn AS Cottrell L Coffman JW Olexa J. Preventive care in Appalachia: use of the theory of planned behavior to identify barriers to participation in cholesterol screenings among West Virginians. J Rural Health. (20016) 22:367–74. 10.1111/j.1748-0361.2006.00060.x 17010036

[36] Jarret T,. Kidding Around Yoga Training Final Report. (2021). Available online at: https://drive.google.com/file/d/1C8uhCDqM3TtoavfloCo5fa5B22MK_z-Z/view (accessed January 10, 2022).

[37] Braun V Clarke V. Using thematic analysis in psychology. Qual Res Psychol. (2006) 3:77–101. 10.1191/1478088706qp063oa32100154

[38] Styck KM Malecki CK Ogg J Demaray MK. Measuring COVID-19 related stress among 4th through 12th grade students. School Psych Rev. (2020) 50:530–45. 10.1080/2372966X.2020.1857658

[39] Shreffler KM Joachims CN Tiemeyer S Simmons WK Teague TK Hays-Grudo J. Childhood adversity and perceived distress from the COVID-19 pandemic. Advers Resil Sci. (2021) 2:1–4. 10.1007/s42844-021-00030-0 33527096 PMC7841380

[40] Fry-Bowers EK. Children are at risk from COVID-19. J Pediatr Nurs. (2020) 53:A10–2. 10.1016/j.pedn.2020.04.026 32386796 PMC7196411

[41] Green P. Risks to children and young people during covid-19 pandemic. BMJ. (2020) 369:m1669. 10.1136/bmj.m1669 32345583

[42] Gazmararian J Weingart R Campbell K Cronin T Ashta J. Impact of COVID-19 pandemic on the mental health of students from 2 semi-rural high schools in Georgia. J School Health. (2021) 91:356–69. 10.1111/josh.13007 33843084 PMC8250377

[43] Pieh C Plener PL Probst T Dale R Humer E. Assessment of mental health of high school students during social distancing and remote schooling during the COVID-19 pandemic in Austria. JAMA Network Open. (2021) 4:e2114866. 10.1001/jamanetworkopen.2021.14866 34181016 PMC8239947

[44] Howarth A Smith JG Perkins-Porras L Ussher M. Effects of brief mindfulness-based interventions on health-related outcomes: a systematic review. Mindfulness. (2019) 10:1957–68. 10.1007/s12671-019-01163-1

[45] Malboeuf-Hurtubise C Taylor G Mageau GA. Impact of a mindfulness-based intervention on basic psychological need satisfaction and internalized symptoms in elementary school students with severe learning disabilities: results from a randomized cluster trial. Front Psychol. (2019) 10:2715. 10.3389/fpsyg.2019.02715 31920787 PMC6915072

[46] Semple RJ. Review: yoga and mindfulness for youth with autism spectrum disorder: review of the current evidence. Child Adolesc Ment Health. (2018) 24:12–8. 10.1111/camh.12295 32677240

